# IgM anti-GM2 antibodies in patients with multifocal motor neuropathy target Schwann cells and are associated with early onset

**DOI:** 10.1186/s12974-024-03090-y

**Published:** 2024-04-17

**Authors:** Kevin Budding, Jeroen W. Bos, Kim Dijkxhoorn, Elisabeth de Zeeuw, Lauri M. Bloemenkamp, Eva M. Zekveld, Ewout J.N. Groen, Bart C. Jacobs, Ruth Huizinga, H. Stephan Goedee, Elisabeth A. Cats, Jeanette H.W. Leusen, Leonard H. van den Berg, C. Erik Hack, W. Ludo van der Pol

**Affiliations:** 1 Center for Translational Immunology, University Medical Center Utrecht, Utrecht, The Netherlands; 2 Department of Neurology and Neurosurgery, University Medical Center Utrecht Brain Center, Utrecht, The Netherlands; 3 Department of Neurology, Erasmus MC, University Medical Center, Rotterdam, The Netherlands; 4 Department of Immunology, Erasmus MC, University Medical Center, Rotterdam, The Netherlands; 5 Department of Neurology, Gelre Hospital, Apeldoorn, The Netherlands

**Keywords:** Multifocal motor neuropathy, Complement, Anti-ganglioside antibodies, IgM anti-GM2, Schwann cells

## Abstract

**Background:**

Multifocal motor neuropathy (MMN) is a rare, chronic immune-mediated polyneuropathy characterized by asymmetric distal limb weakness. An important feature of MMN is the presence of IgM antibodies against gangliosides, in particular GM1 and less often GM2. Antibodies against GM1 bind to motor neurons (MNs) and cause damage through complement activation. The involvement of Schwann cells (SCs), expressing GM1 and GM2, in the pathogenesis of MMN is unknown.

**Methods:**

Combining the data of our 2007 and 2015 combined cross-sectional and follow-up studies in Dutch patients with MMN, we evaluated the presence of IgM antibodies against GM1 and GM2 in serum from 124 patients with MMN and investigated their binding to SCs and complement-activating properties. We also assessed the relation of IgM binding and complement deposition with clinical characteristics.

**Results:**

Thirteen out of 124 patients (10%) had a positive ELISA titer for IgM anti-GM2. Age at onset of symptoms was significantly lower in MMN patients with anti-GM2 IgM. IgM binding to SCs correlated with IgM anti-GM2 titers. We found no correlation between IgM anti-GM2 titers and MN binding or with IgM anti-GM1 titers. IgM binding to SCs decreased upon pre-incubation of serum with soluble GM2, but not with soluble GM1. IgM anti-GM2 binding to SCs correlated with complement activation, as reflected by increased C3 fixation on SCs and C5a formation in the supernatant.

**Conclusion:**

Circulating IgM anti-GM2 antibodies define a subgroup of patients with MMN that has an earlier onset of disease. These antibodies probably target SCs specifically and activate complement, similarly as IgM anti-GM1 on MNs. Our data indicate that complement activation by IgM antibodies bound to SCs and MNs underlies MMN pathology.

**Supplementary Information:**

The online version contains supplementary material available at 10.1186/s12974-024-03090-y.

## Background

Multifocal motor neuropathy (MMN) is a rare, chronic motor neuropathy characterized by slowly progressive asymmetric weakness of distal limbs [1–4], that responds to treatment with intravenous or subcutaneous immunoglobulins (IVIg; ScIg) [5, 6]. (Multi)focal motor conduction block with normal sensory function is considered the hallmark of MMN, but imaging studies have shown a more generalized pattern of nerve pathology [7–9]. Serum from patients with MMN often contains IgM antibodies against ganglioside GM1 and occasionally GM2 [3, 10, 11]. The pathophysiological mechanisms underlying MMN are incompletely understood due to the few pathological studies performed in MMN and the lack of a representative animal model [12], but the available evidence suggests immune-mediated abnormalities of (perinodal and perisynaptic) Schwann cells (SCs), myelin sheath and the (peri)nodes of Ranvier [10, 13, 14].

GM1 is a glycosphingolipid that is highly expressed in perinodal regions of peripheral nerves and a target for antibodies, found in patients with MMN and acute motor axonal neuropathy (AMAN) [11]. Anti-GM1 IgM binds to axons and neurites of induced pluripotent stem cell (iPSC) derived motor neurons (MNs) and induces cellular damage through the activation of the classical complement pathway [10, 14]. Hence, complement activation by IgM anti-GM1 antibodies may underlie disease progression and permanent weakness due to accumulating axonal damage [15]. Higher titers of anti-GM1 are associated with both more complement deposition in vitro and more pronounced weakness in patients [3, 16, 17].

Initial discrepancies of anti-GM1 IgM prevalence reports in MMN were caused by differences in methodology [18], but recent studies showed that IgM anti-GM1 are present in serum of approximately 50% of patients [3, 4, 12, 13]. This is an underestimation due to limited sensitivity of detection techniques [10], but it is likely that serum from a subgroup of patients with MMN does not contain IgM anti-GM1, but IgM auto-antibodies with other specificities, such as NS6S heparin disaccharide [19] and other gangliosides, such as GM2, GD1b, and GD1a [3, 20–22].

We previously described the pathogenic effects of MMN-associated antibodies using an iPSC-MN model [10, 14]. The goal of this study was to study binding of IgM antibodies against gangliosides using a SC-line as well as iPSC-derived MNs and their potency to activate complement in a cohort of 124 well characterized patients with MMN.

## Methods

### Standard protocol approvals, registrations, and patient consents

The Ethics Committee of the University Medical Center Utrecht approved the collection of patient sera as part of a national cross-sectional study (UMCU, METC protocol nr: 14–528) [4]. Written informed consent was obtained from all study participants prior to inclusion in this study.

### Study populations

All patients with MMN had been diagnosed at the outpatient clinic of the UMCU and met the 2010 EFNS diagnostic criteria for definite, probable or possible MMN [23]. We only included patients of whom clinical data were available. We obtained serum samples of healthy controls (HC) through the in-house donor facility of the UMCU. Serum samples of all subjects were heat-inactivated for 30 min at 56 °C and stored in aliquots at -80 °C until used.

### Clinical data

We retrieved clinical data from the UMCU MMN database. This registry contains data collected during the 2007 and 2015 Dutch national combined cross-sectional and follow-up studies on MMN, complemented with data from patients’ UMCU patient files [3, 4, 24]. Age at onset was defined as the age at which a patient first noticed signs of muscle weakness, and disease duration was defined as the time that lapsed since disease onset. Nerve conduction studies (NCS) were done as described previously [4, 24]. We recorded the presence of abnormal brachial plexus MR imaging (nerve thickening or nerve hyperintensity), postural hand tremor and vibration sense abnormalities [3, 4]. We calculated an MRC sum score (MRCss) of shoulder abduction, elbow, wrist and finger flexion and extension, finger spreading, hip and knee flexion, knee extension and foot dorsal and plantar flexion bilaterally as a measure for total muscle strength (maximum score 130) at patients’ first visit to our hospital [25]. The difference in MRCss (ΔMRCss) as measured during the Dutch national cross-sectional studies on MMN in 2007 and 2015 was used as a measure of disease progression [3, 4]. We listed the presence of IgG and/or IgM monoclonal gammopathy as determined in serum, cerebrospinal fluid (CSF) analysis results and anti-GM1 and anti-GM2 IgM antibody status, which were determined by ELISA as described previously [3, 26]. For IVIg analyses, we determined patients’ IVIg treatment status and IVIg dosage in grams/month.

### Cell culture

The iPSC-derived model for MMN was modified from protocols described previously [10, 14]. Human SC-line sNF96.2 (derived from a malignant peripheral nerve sheath tumor) was obtained from ATCC (CRL-2884) and cultured in T75 or T175 flasks (Greiner) in DMEM medium (Life Technologies) supplemented with 100 U/mL penicillin (Life Technologies), 100 µg/µL streptomycin (Life Technologies), and 10% v/v (volume/volume) fetal calf serum (FCS, Bodinco) at 37 °C and 5%, v/v, CO_2_. Cells were passaged at 80% confluency, first washed in PBS, following detachment using Accutase cell detachment solution (Sigma-Aldrich). Cell numbers and viability (typically > 80%) were assessed via trypan blue (Sigma-Aldrich) exclusion assay using an automated cell counter (Countess, Invitrogen).

### Flow cytometry

sNF96.2 SCs were transferred to V-bottom plates (Greiner) at a density of 50,000 cells/well. To assess IgM binding, cells were opsonized with heat-inactivated (HI) MMN serum or HC serum (1:20 diluted in veronal buffer (VB, Lonza)) for 1 h at room temperature (RT). Between every incubation step, cells were washed with 100µL FACS buffer (FB, which is phosphate buffered saline, pH 7.4 (PBS, Sigma)-0.1%, w/v, bovine serum albumin (BSA, BSA Fraction V, Roche)-0.01%, w/v, sodium azide) and centrifuged for 5 min at 125*g*. Next, cells were stained (20 µl, diluted in FB, 45 min on ice in the dark) with a primary detection antibody (goat anti-human IgM biotin, 1:50, Sigma) followed by incubation with a secondary detection antibody (streptavidin-APC, 1:100, ThermoFisher). To correct for day-to-day variation, the mean fluorescent intensity (MFI) of each sample was divided by the average MFI of a set of HC sera (*n* = 6) that we tested simultaneously and expressed as fold change (FC). To assess IgM anti-GM1 and/or anti-GM2 antibody specificity, MMN patient sera were pre-incubated with soluble GM1 (Enzo Life Sciences) or GM2 (Sigma) at 100 µg/µL for 30 min at RT, prior to opsonization. The % inhibition of IgM binding was defined in ELISA as the reduction in antibody activity defined by OD in a serum sample preincubated with GM1/GM2 compared to a serum sample without such preincubation. To evaluate complement activation by bound IgM antibodies, following opsonization with MMN patient or HC serum cells were incubated with 5%, v/v, pooled complement active serum (InnovativeResearch), diluted in VB. Fixation of C3 to the cells was then measured by a subsequent inhibition with mouse anti-human C3 biotin (1:25 in FB, LSBio). Cells were analyzed using flow cytometry (FACS Canto II, and accompanying software, FACS DIVA, BD Biosciences).

### Anti-GM2 specific ELISA

To assess GM2 specificity, we utilized a modified version of the previously published anti-GM2 ELISA [3, 26]. Wells of a MaxiSorp plate (NUNC) were coated with 70µL 0.1 µg/mL GM2 (Sigma), diluted in methanol, and left to evaporate O/N in a laminar flow. Wells were blocked with 200 µL 1% BSA-PBS for 1 h at RT. Patient sera were diluted 1:200 in 1% BSA-PBS, either or not pre-incubated with GM1 or GM2 (50 µg/µL for 30 min at RT), and incubated in GM2 coated wells 1 h at RT. Next, wells were washed 3 times with PBS, and IgM binding was detected using goat anti-human IgM (Sigma, 70µL 1:10000 in 1% BSA-PBS, 1 h at RT), followed by a 3-time wash in PBS, and incubation with streptavidin-POD (Sigma, 70µL 1:1000 in 1% BSA-PBS, 30 min at RT), and a final 3-time wash in PBS. For detection 100µL TMB (Invitrogen) was added to each well. Finally, the reaction was stopped using 1 M HCL (Fisher Chemical). All measurements were conducted in triplicate and OD_450 nm_ (read-out at a wave length of 450 nm) was analyzed using a SpectraMax M3 (Molecular devices). The d-OD_450nm_ was obtained by subtracting the OD_450nm_ from an uncoated well of the respective OD_450nm_ of a GM2 coated well. The % inhibition of IgM binding was calculated by setting the OD_450nm_ of the serum sample without GM1/GM2 preincubation at 0% inhibition.

### Microscopy and live cell complement activation

sNF96.2 SCs were seeded (50.000 cells/well) on coverslips (VWR) in a 24-well plate (Greiner) for 2 days prior to experimental analysis. Cells were opsonized with HI MMN patient serum (150 µL, 1:50 in VB) for 1 h at RT. Next, 150 µl 15% pooled complement active serum (pre-incubated with complement inhibitors for 15 min at RT when indicated) was added to each well and incubated for 1 h at 37 °C. Supernatant was collected after incubation with complement-active serum for the measurement of complement activation products. For microscopy, cells were fixed in 4% paraformaldehyde (Klinipath) 10 min at RT, the coverslips were removed from the 24 wells plate, washed with PBS and quenched for autofluorescence using NH_4_Cl (5 min RT). Subsequent incubation steps were performed top-down in 100 µL droplets on parafilm. Coverslips were blocked in 2% BSA-PBS for 1 h RT, and subsequently stained with a primary (biotinylated goat anti-human C3, 1:2000, MyBioSource) and secondary detection antibody (Streptavidin APC, 1:100, eBioscience), both diluted in 2% BSA-PBS, for 1 h at RT in the dark and washed with PBS. After the last antibody incubation step coverslips were washed in PBS and distilled water (Milli-Q). After removal of excessive liquid, the coverslip was mounted on an object glass in 7µL ProLong Diamond Antifade Mountant with DAPI (Invitrogen) and dried overnight at RT. Samples were analyzed at 20x magnification using a Zeiss Z1 microscope (Carl Zeiss Microscopy) with Colibri LEDs and the following settings: 25% LED 400 ms for Alexa Fluor 488, 25% LED 100 ms for APC, 25% LED 50 ms for DAPI. To prevent bias, 4 pictures were taken throughout the image field. Pictures were exported in single and merged channel to non-compressed TIFF-format using ZEN 2 software (Carl Zeiss Microscopy) and mean grey values were calculated for each single channel using ImageJ (Fiji 1.53).

### Other analyses

C5a in the supernatant samples after complement activation was measured with the Human Complement Component C5a DuoSet ELISA (R&D Systems) according to manufacturer’s instructions. For all complement read-outs, values were normalized by calculating the FC relative to the non-opsonized serum control from each experiment set at 1.

### Statistical analysis

GraphPad Prism 9 was used for data analysis and visualization of experimental data. Correlation analyses were conducted using the Spearman’s rank correlation coefficient with the following r_s_ grading: 0.00-0.10 negligible; 0.10–0.39 weak; 0.40–0.69 moderate; 0.70-1.00 strong. Clinical data were analyzed using R version 4.2.0. We compared categorical data using a Chi-squared test. Continuous variables were compared between groups using a Student’s t-test or Mann-Whitney U test, as appropriate. Multiple-group comparisons were performed using a Kruskal-Wallis test with Dunn’s test as a post-hoc analysis. In analyses concerning IVIg dosage (grams/month) and MRC sum score, i.e., the MRCss at patients’ first visit and the change in muscle strength between 2007 and 2015 which we termed ΔMRCss, comparisons between groups were corrected for disease duration using a linear regression model. Analyses involving patients’ MRCss at their first visit to the UMCU were performed in IVIg treatment-naïve patients only. For nerve conduction comparisons, we included a subset of patients whose samples were used in the complement activation assay (see above), of whom detailed nerve conduction data were available. A *p*-value < 0.05 was considered statistically significant. When appropriate, we corrected *p*-values for multiple testing using the Bonferroni method.

## Results

### Study population

**Fig. 1:**
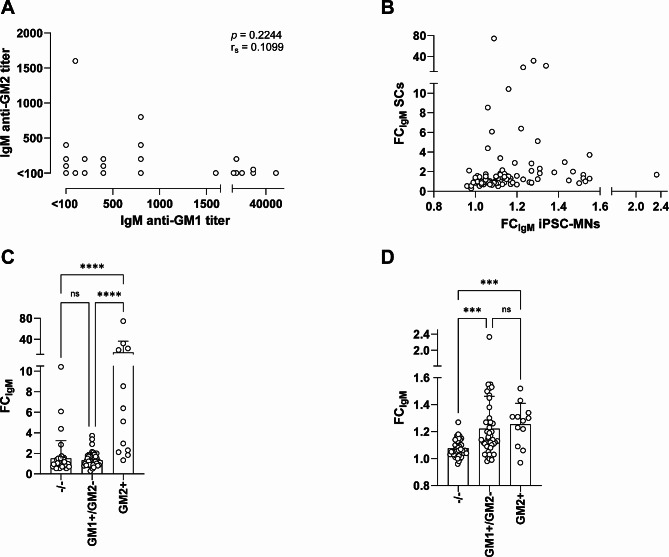
IgM titers and MMN patient serum-derived IgM binding to SCs and iPSC-MNs. (**A**) Correlation between IgM anti-GM1 and IgM anti-GM2 titers as determined via ELISA. (**B**) Sera from 98 MMN patient sera were screened for IgM binding to SCs using flow cytometry. IgM binding is depicted as fold-change (FC) compared to the mean IgM binding of 6 healthy control sera tested in the same assay on the y-axis. IgM binding to iPSC-MNs incubated with MMN sera using microscopy. IgM binding is expressed as FC similarly as in (**A**). (**C**) Stratification of MMN patients by IgM anti-ganglioside antibody status determined with ELISA reveals higher IgM binding to SCs in GM2 + versus GM1+/GM2- and -/- patients (Kruskal-Wallis, post-hoc Dunn’s multiple comparisons test). (**D**) Stratification of MMN patients by IgM anti-ganglioside antibody status indicates higher IgM binding to iPSC-MNs in GM1 + versus GM1- patients. Kruskal-Wallis, post-hoc Dunn’s multiple comparisons test. **** *p* < 0.0001; *** *p* < 0.001; FC: fold change; ns: non-significant

We included 124 patients with MMN in the study. We previously assessed anti-GM1 and anti-GM2 titers with ELISA in serum samples from 87 patients collected during the 2007 cross-sectional study [24], and we used the same methodology for 37 additional patients in the present study, whose samples were collected during the 2015 cross-sectional study [3, 26]. Clinical characteristics are summarized in Table 1. IgM anti-GM1 and anti-GM2 antibodies as detected by ELISA were present in 58% and 10% of the patients, respectively, while 39% was negative for either one of these antibodies. IgM anti-GM1 and IgM anti-GM2 titers did not correlate (Fig. 1A; r_s_=0.1099, *p* = 0.2244), suggesting that these are two different types of antibodies rather than one cross-reacting antibody.

**Table 1:** Baseline characteristics of 124 patients with MMN included in this study

	MMN	IgM binding experiments		*p*
	*N* = 124	Serum available (*n* = 98)	No serum available (*n* = 26)	
Male sex^$^	93 (75)	76 (77)	17 (65)	0.31
Age at onset (years)^#^	42 (16)	41 (13)	43 (24)	0.45
Diagnostic delay (years)^#^	6.8 (12)	7.5 (16)	5.7 (5.3)	0.22
MMN EFNS 2010 diagnosis^$^				0.69
Definite	88 (72)	68 (70)	20 (77)	
Probable	26 (21)	21 (22)	5 (19)	
Possible	9 (7)	8 (8)	1 (4)	
IgM Anti-GM1 positive^$^	72 (58)	52 (53)	20 (77)	0.049^*^
IgM Anti-GM2 positive^$^	13 (10)	11 (11)	2 (8)	0.60
IVIg treatment at sampling^$^	-	85 (87)	-	
IVIg dosage (grams/week) ^#^	-	13	-	

A separate column shows the baseline characteristics of 98 patients with MMN of whom serum was available for SC IgM binding experiments. Comparisons were made between this group of 98 patients and the remainder of patients (n = 26), the p-values of which are shown in the right column. A separate column shows the baseline characteristics of 98 patients with MMN of whom serum was available for SC IgM binding experiments. Comparisons were made between this group of 98 patients and the remainder of patients (n = 26), the p-values of which are shown in the right column

^*^Statistically significant at p-value < 0.05

^$^ Values displayed as n (%)

^#^ Values displayed as median (IQR)

SC Schwann cell, EFNS European federation of neurological societies, IVIg intravenous immunoglobulin, MMN Multifocal motor neuropathy

We next investigated specific clinical characteristics of patients with IgM anti-GM2. To this end, we stratified patients by the presence or absence of IgM anti-GM1 and GM2: IgM anti-GM2 positive, either or not with anti-GM1 (group A); IgM anti-GM1 positive and anti-GM2 negative (group B); and double negative (group C). We did not analyze patients with IgM anti-GM2 without GM1 antibodies (*n* = 4) separately due to small numbers. Comparisons between groups are shown in Table 2.

**Table 2:** Clinical parameters of patients with MMN (*N* = 124), stratified by IgM anti-GM1 and anti-GM2 antibody status

	Patient groups			*p*-values	
	(A) Anti-GM2 +	(B) Anti-GM1+	(C) Anti-GM2/GM1-	A vs. B	A vs. C
	*N* = 13	*N* = 63	*N* = 48		
IgM anti-GM1 IgM positive (n (%))	9 (69)	63 (100)	0 (0)	-	-
IgM anti-GM2 IgM positive (n (%))	13 (100)	0 (0)	0 (0)	-	-
Male sex (n (%))	10 (77)	47 (75)	36 (75)	1.00	1.00
EFNS MMN diagnosis (n (%))				0.88	0.54
Definite	10 (77)	42 (67)	36 (77)		
Probable	3 (23)	16 (25)	7 (15)		
Possible	0 (0)	5 (8)	4 (8)		
Age at onset (median years (IQR))	31 (16)	43 (13)	42 (14)	**0.015** ^*^	**0.034** ^*^
Inclusion MAIN 2007 (n (%))	12 (92)	50 (79)	25 (52)	-	-
Inclusion MAIN 2015 (n (%))	10 (77)	43 (68)	41 (85)	-	-
Inclusion MAIN 2007 & MAIN 2015 (n (%))	9 (69)	30 (47)	20 (42)	-	-
Disease duration (median months (IQR))					
At first UMCU visit	57 (96)	49 (80)	32 (63)	0.36	**0.037** ^*^
MAIN 2007	216 (120)	132 (141)	120 (96)	0.13	**0.042** ^*^
MAIN 2015	308 (170)	216 (182)	166 (122)	0.16	**0.008** ^*^
Nerve conduction studies at diagnosis					
Conduction block (n (%))	12 (93)	50 (93)	43 (96)	1.00	0.54
Definite conduction block (n (%))	9 (69)	34 (63)	36 (80)	0.76	0.46
Axonal damage (n (%))	7 (54)	24 (44)	13 (29)	0.55	0.11
Nerves with CB (median (range))	2 (0–9)	3 (0–12)	3 (0–14)	0.94	0.90
Nerves with definite CB (median (range))	1 (0–5)	1 (0–5)	1 (0–8)	0.68	0.84
Nerves with axonal damage (median (range))	1 (0–7)	0 (0–7)	0 (0–3)	0.73	0.31
IVIg treatment (n (%))					
At first UMCU visit	5 (42)	16 (25)	4 (9)	0.30	**0.015** ^*^
MAIN 2007	10 (83)	39 (78)	17 (68)	1.00	0.44
MAIN 2015	10 (100)	39 (91)	15 (75)	1.00	0.14
IVIg dosage (median gr/month (IQR))					
MAIN 2007	18 (9)	14 (8)	12 (7)	0.33	0.16
MAIN 2015	20 (8)	15 (10)	10 (7)	0.85	0.08
Monoclonal gammopathy (n (%))					
IgM	1 (8)	6 (10)	3 (7)	1.00	1.00
IgG	0 (0)	2 (3)	2 (5)	1.00	1.00
CSF studies performed (n (%))	2 (15)	15 (26)	15 (31)	0.72	0.48
CSF elevated protein level	2 (100)	15 (100)	9 (60)	1.00	0.51
CSF leukocytosis	0 (0)	0 (0)	1 (14)	-	-
Brachial plexus MRI abnormalities (n (%))	7 (78)	17 (45)	10 (33)	0.14	**0.026** ^*^
MRC sum score (median (IQR))					
At first UMCU visit	115 (4)	121 (9)	124 (8)	0.12	**0.0003** ^*^
ΔMRCss (MAIN 2007–2015)	-8 (9)	-11 (10)	-4 (7)	0.41	0.13
Postural tremor (n (%))	8 (80)	27 (64)	25 (63)	0.47	0.46
Sensory abnormalities (n (%))					
Hypesthesia/paresthesia	4 (33)	3 (6)	4 (16)	**0.023** ^*^	0.39
Vibration sense abnormalities	3 (25)	13 (26)	3 (12)	1.00	0.37

^*^ Statistically significant at p-value < 0.05

All analyses concerning IVIg dosage and MRC sum scores were corrected for disease duration. Analyses concerning the MRC sum score at patients’ first visit to the UMCU were performed in treatment-naïve patients only

CB = conduction block, CSF = cerebrospinal fluid, IVIg = intravenous immunoglobulins, MMN = multifocal motor neuropathy, MRCss = MRC sum score, MRI = magnetic resonance imaging

Patients with IgM anti-GM2 had a significantly lower age at onset of disease, with a median difference of 12 years. Patients with IgM anti-GM2 antibodies had significantly lower MRCss than patients without antibodies, but this difference must probably be attributed to the concomitance of IgM anti-GM1, which was associated with more pronounced weakness in a previous study [3]. Indeed, comparison of patients with only IgM anti-GM1 antibodies and those without antibodies showed significantly lower MRCss at first visit Utrecht (*p* = 0.046) [4, 24]. Finally, reported sensory symptoms such as hypesthesia or paresthesia were more frequent in the group with IgM anti-GM2 (group A) (33% vs. 6%, *p* = 0.023).

Finally, we compared available nerve conduction studies performed at diagnosis of 16 patients with MMN, of whom 7 had IgM anti-GM2 antibodies. Results are shown in Table 3. There were no differences in nerve conduction velocities of motor nerves between patients with or without IgM anti-GM2 antibodies. Patients with IgM anti-GM2 antibodies did not have lower sensory nerve action potential (SNAP) amplitudes of altered sensory conduction velocities as compared to patients without IgM anti-GM2 antibodies. F-wave latencies in all investigated nerves were comparable between groups.

**Table 3:** Comparing detailed NCS data between patients with MMN with IgM anti-GM2 antibodies (*n* = 7) and without (*n* = 9)

CMAPs/SNAPs/F-waves		GM2 +	GM2 -	*p*	Velocities (m/s)	GM2 +	GM2 -	*p*
**Median nerve**								
DML		4.2 (0.65)	4.1 (0.83)	0.86	-	-	-	-
CMAP	Wrist	8.7 (4.2)	7.5 (11.3)	0.8	-	-	-	-
	Elbow	6.0 (7.2)	6.0 (7.8)	0.92	Forearm	48 (15.5)	51 (19.8)	0.85
	Axilla	2.9 (4.4)	6.0 (6.9)	0.46	Upper arm	50 (11)	55 (20.5)	0.40
	Erb’s point	3.3 (6.4)	6.5 (6.5)	0.59	Erb’s point	68 (23.8)	67 (10)	0.63
SNAP	Wrist	17.1 (13.2)	20.8 (15.9)	0.88	SNAP velocity	52 (3)	55 (7)	0.25
F-wave latency		35.1 (15.1)	33.2 (7.1)	0.70	-	-	-	-
**Ulnar nerve**								
DML		3.5 (1.1)	3.4 (0.53)	0.55	-	-	-	-
CMAP	Wrist	6.4 (3.0)	7.9 (9.7)	0.58	-	-	-	-
	Sulcus (distal)	2.9 (4.9)	7.8 (9.0)	0.58	Forearm	52 (25.8)	54 (9)	0.31
	Sulcus (proximal)	3.0 (5.5)	7.5 (9.0)	0.49	Sulcus	47 (12.3)	50 (12.3)	0.43
	Axilla	2.7 (4.7)	4.4 (6.9)	0.47	Upper arm	49 (25)	56 (33.5)	0.80
	Erb’s point	1.9 (4.1)	4.5 (6.2)	0.42	Erb’s point	63 (23)	64 (12)	0.42
SNAP	Wrist	25 (22.9)	10 (6.7)	**0.0074** ^*^	SNAP	51 (2.8)	48 (6.3)	0.38
F-wave latency		36.1 (26.9)	31.7 (7.3)	0.65	-	-	-	-
**Radial nerve**								
SNAP	Wrist	17 (15.4)	14.9 (13.5)	0.27	SNAP velocity	53 (4)	52 (4.3)	0.66
**Deep peroneal nerve**								
DML		4.1 (0.7)	5.1 (1.9)	**0.007**	-	-	-	-
CMAP	Ankle	3.6 (5.6)	3.7 (4.1)	0.98	-	-	-	-
	Fibular head (distal)	2.8 (4.4)	2.9 (4.2)	0.63	Lower leg	46 (7)	42 (6)	0.26
	Fibular head (proximal)	2.7 (3.7)	2.8 (4.1)	0.63	Fibular head	46 (5)	47 (6)	0.79
F-wave latency		57.3 (11.9)	59.7 (9.3)	0.24	-	-	-	-
**Tibial nerve**								
DML		5.1 (1.0)	4.9 (1.8)	0.65	-	-	-	-
CMAP	Ankle	6.5 (6.2)	9 (8.2)	0.65	-	-	-	-
	Knee	3.4 (5.4)	4.4 (6.2)	0.59	Lower leg	44 (4)	45 (2)	1.00
F-wave latency		58.9 (18)	60.3 (5.0)	0.89	-	-	-	-
**Sural nerve**								
SNAP		9 (3.5)	12 (9.5)	0.52	SNAP velocity	46 (8)	48 (7)	0.28

^*^ Statistically significant at p-value < 0.05

CMAP Compound muscle action potential, DML Distal motor latency, MMN Multifocal motor neuropathy, NCS Nerve conduction studies, SNAP Sensory nerve action potential

### IgM anti-GM2 antibodies from patients with MMN bind to SCs

To investigate binding of IgM anti-GM1 or -GM2 to SCs and iPSC-MNs, we used 98 available sera. Apart from a minor difference in IgM anti-GM1 positivity, clinical characteristics of these patients were similar to those of the other 26 patients (Table 1). FC of IgM antibody against SCs in MMN patient’s sera varied between 0.28 and 74.54. Moreover, IgM anti-SC FC differed from IgM anti-iPSC-MNs measured and expressed in a similar way (Fig. 1B).

**Fig. 2:**
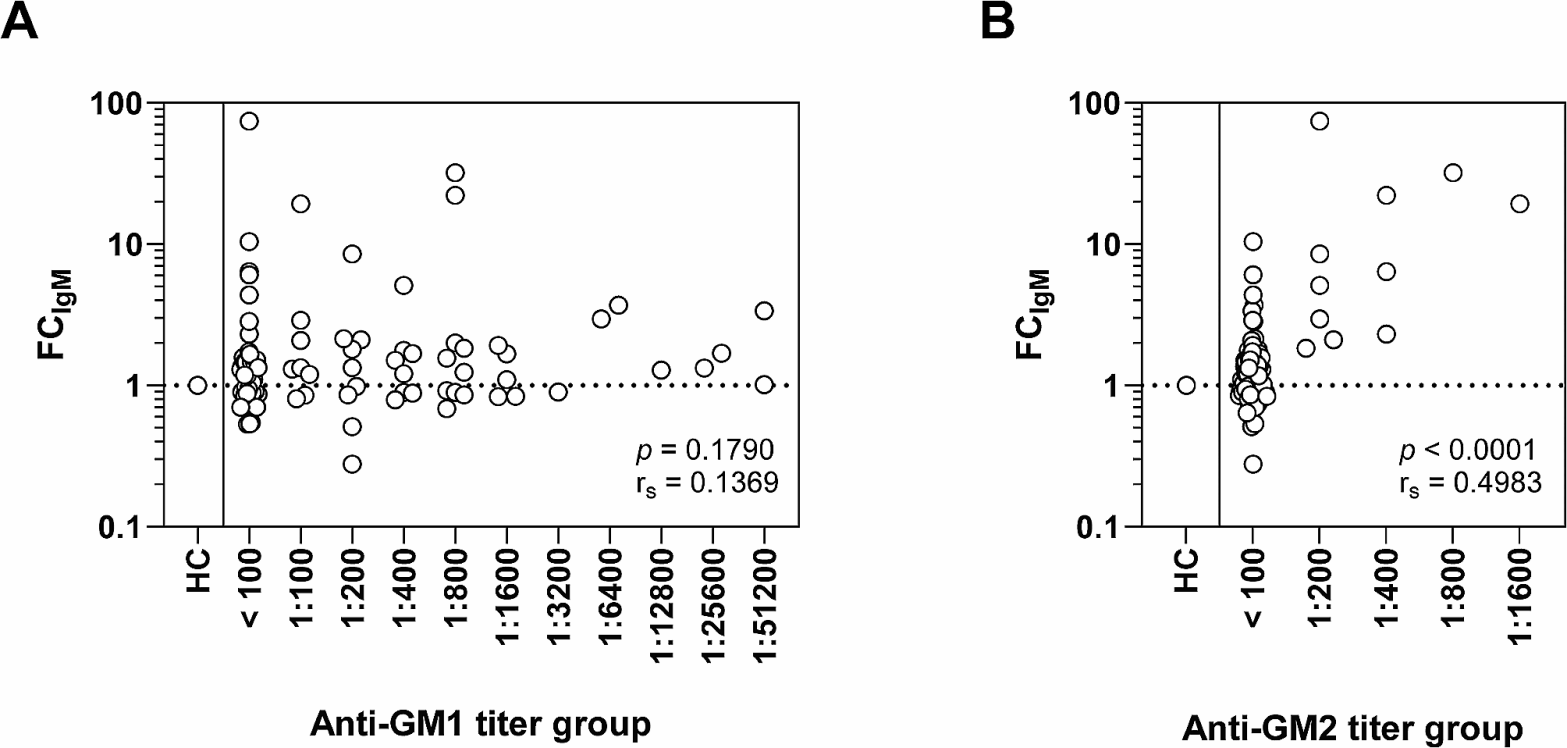
IgM binding to SCs stratified by IgM anti-GM2 and anti-GM1 antibody titer. (**A**) IgM binding to SCs stratified for IgM anti-GM1 titer. No correlation was found between FC_IgM_ and IgM anti-GM1 titers (Spearman’s rho r_s_=0.1369, *p* = 0.1790). (**B**) IgM binding to SCs stratified for IgM anti-GM2 antibody titer group shows a moderate correlation between IgM binding (FC_IgM_) and anti-GM2 titer (Spearman’s rho r_s_=0.4983, *p* < 0.0001). FC: fold change

Next, we investigated differences in IgM binding to SCs and iPSC-MNs among patients stratified by their IgM anti-GM1/2 status as determined with ELISA. Regarding IgM binding to SCs, we observed a significantly higher FC for patients positive for anti-GM2 compared to anti-GM2 negative patients. FC of IgM did not differ between patients with or without IgM anti-GM1 (Fig. 1C, and Supplemental Fig. 1). In contrast, binding of IgM to the iPSC-MNs was significantly higher in the patients who had IgM anti-GM1 antibodies compared to patients negative for these antibodies (Fig. 1D). FC on SCs moderately correlated with IgM anti-GM2 titers measured with ELISA (r_s_ = 0.4983, *p* < 0.0001), but not with IgM anti-GM1 titers (Fig. 2A and B).

### IgM binding of MMN patients to SCs is GM2 specific

**Fig. 3:**
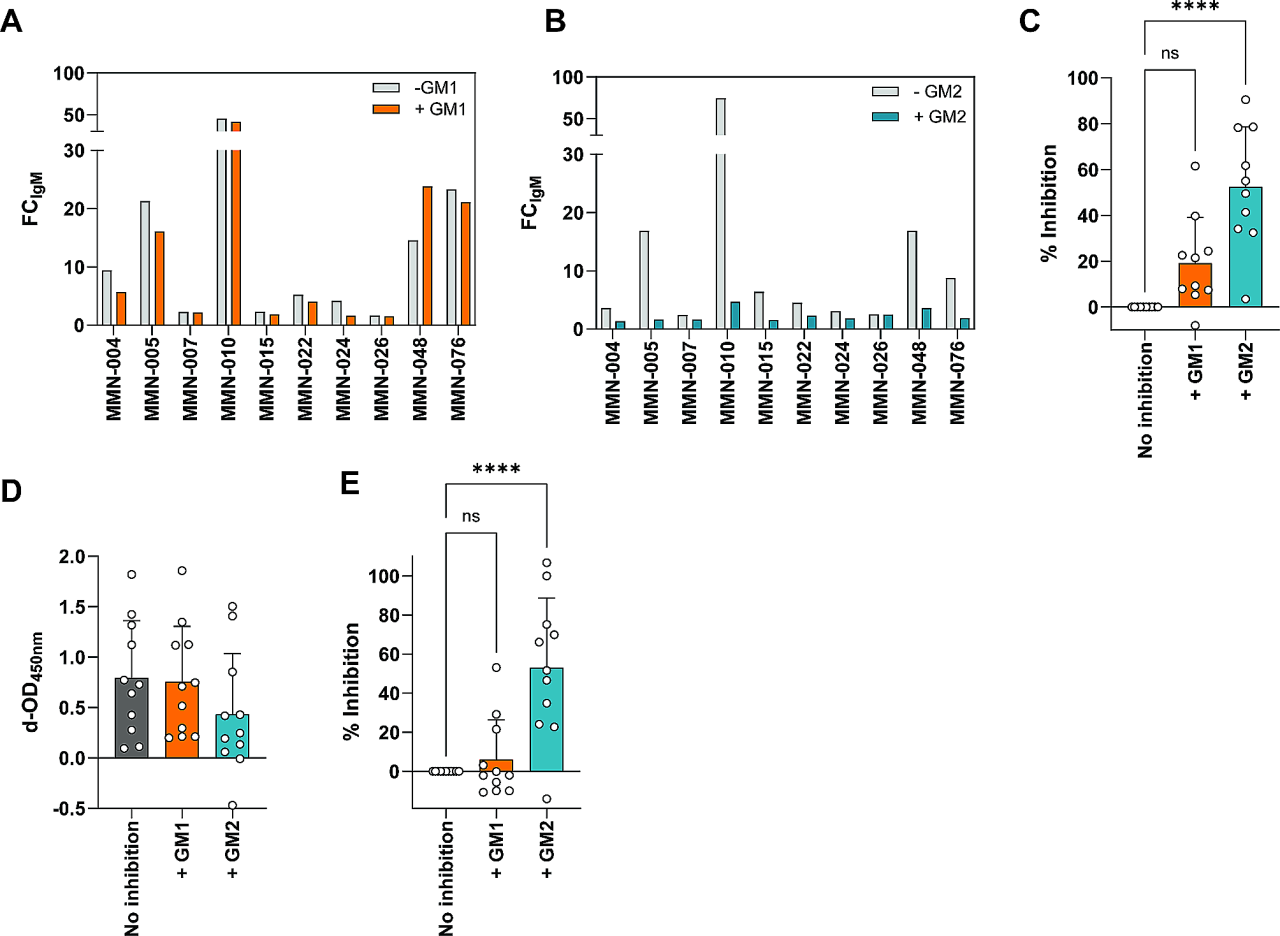
MMN patient-derived IgM binding to SCs is GM2 specific. Sera from MMN patients with IgM anti-GM2 were selected, incubated with soluble GM1 or GM2 and tested for IgM binding to SCs and in the anti-GM2 ELISA. (**A**) FC_IgM_ binding on SCs before (grey bars) and after (orange bars) pre-incubation with GM1. (**B**) FC_IgM_ binding on SCs before (grey bars) and after (blue bars) pre-incubation with GM2. (**C**) Quantification of flow cytometric results depicted in (**A** and **B**) as % inhibition. IgM binding is significantly decreased upon pre-incubation with GM2, whereas pre-incubation with GM1 only does not significantly decrease IgM binding. (**D**) Anti-GM2 specific ELISA showing IgM binding (in d-OD_450nm_) without (grey bar) or with pre-incubation with GM1 (orange bars) or GM2 (blue bars) using IgM anti-GM2 positive MMN patient sera. (**E**) Quantification of ELISA results depicted in (**D**) as % inhibition. Pre-incubation with GM2 significantly lowers IgM binding. Mean + SD, Kruskal-Wallis, post-hoc Dunn’s multiple comparisons test. **** *p* < 0.0001; ns: non-significant

To confirm anti-GM2 antibody specific binding to SCs, we pre-incubated MMN sera positive for anti-GM2 IgM with soluble GM1 and GM2 and tested residual IgM binding to membrane bound or solid-phase GM2 using flow cytometry and a GM2-specific ELISA. Pre-incubation of anti-GM2-positive MMN patient serum with soluble GM1 resulted in a modest reduction in FC of IgM binding to SCs (Fig. 3A), whilst pre-incubation with soluble GM2 strongly reduced residual FC IgM binding (Fig. 3B). When plotted as % inhibition of IgM binding, setting the non-treated serum sample at 0% inhibition, we observed a significant inhibition of IgM binding to SCs upon pre-incubation of the IgM anti-GM2 positive sera with soluble GM2. Under the same conditions, we did not observe significant inhibition upon pre-incubation of these sera with soluble GM1 (Fig. 3C). Upon preincubation of anti-GM2-positive MMN patient sera with soluble GM1, OD_450nm_ did not decrease, whereas pre-incubation with soluble GM2 resulted in a significant inhibition of IgM binding (Fig. 3D and E) thereby confirming the flow cytometry results and specificity of the IgM antibodies towards GM2 expressed on SCs.

### IgM anti-GM2 binding on SCs results in complement activation

**Fig. 4:**
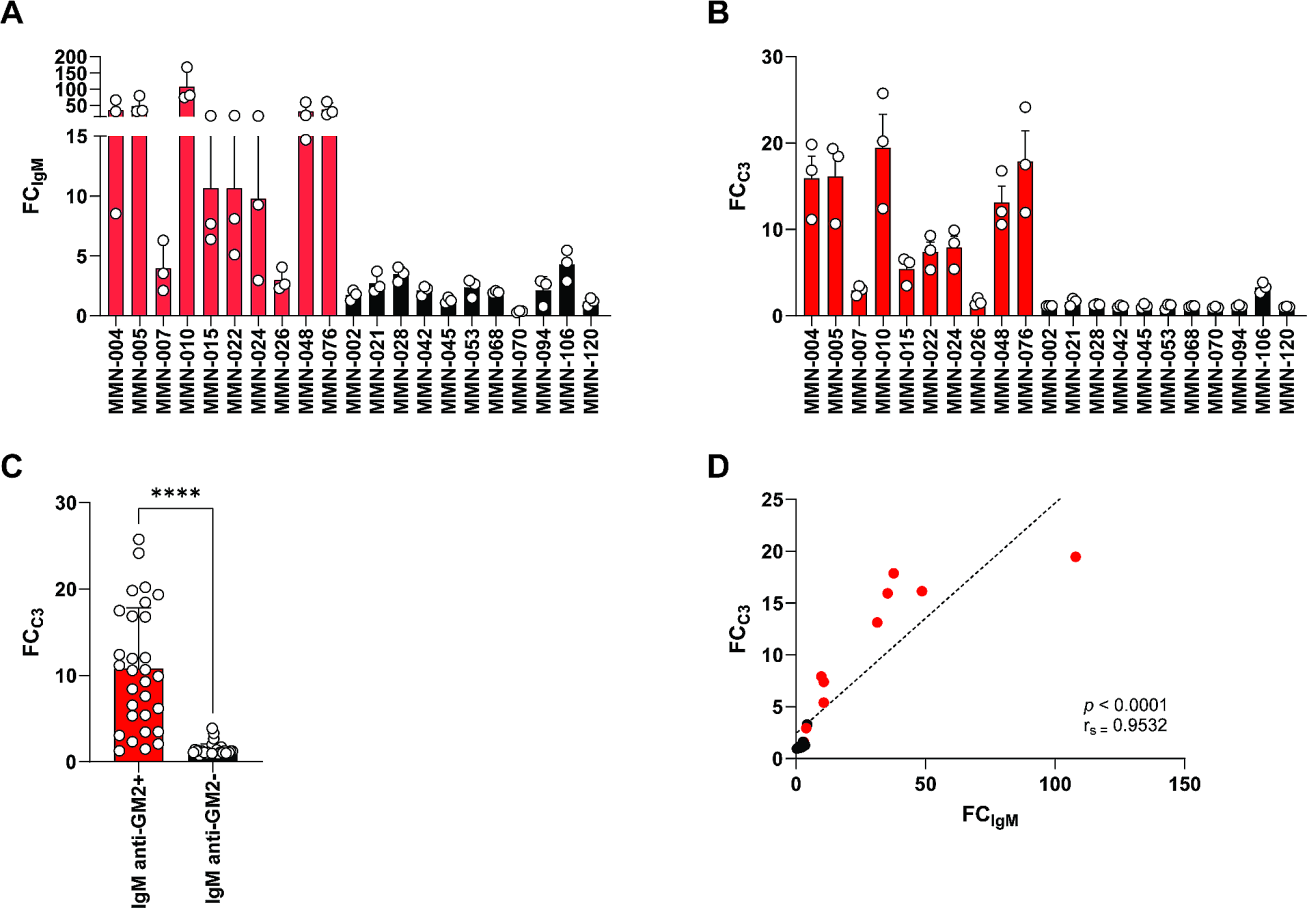
IgM anti-GM2 binding on SCs results in complement activation. IgM binding (FC_IgM_, **A**) and C3 fixation (FC_C3_, **B**) to SCs opsonized with IgM anti-GM2 positive (red bars) or negative (black bars) MMN patient serum and incubated with fresh serum as complement source. Opsonization with anti-GM2 MMN patient serum results in increased IgM binding and increased complement activation. Data are mean + SD of different assays (**C**) Pooled results of FC_C3_ data depicted in (**B**), Mann-Whitney test. (**D**) Highly significant strong correlation between IgM anti-GM2 binding to SCs and subsequent complement activation. Red dots IgM anti-GM2 + sera, black dots IgM anti-GM2- sera. Mean + SD, Spearman’s rho r_s_=0.9532, *p* < 0.0001. **** *p* < 0.0001; FC: fold change

Complement activation by IgM anti-GM1 antibody bound to MNs is probably a major mechanism in the pathogenesis of MMN [3, 10, 16]. We therefore investigated whether IgM anti-GM2 antibody binding induced complement activation on SCs. SCs were incubated with HI sera from patients with MMN either or not positive for IgM anti-GM2, and with fresh pooled human serum as a source of active complement. Since the majority of IgM anti-GM2 positive sera also contained IgM anti-GM1, we compared sera with IgM anti-GM2 to sera without anti-GM2 IgM, with and without IgM anti-GM1. A detailed overview of antiganglioside antibody titers for each patient can be found in Supplemental Table 1. Results were expressed similarly as for IgM binding (Fig. 4A). C3 fixation (Fig. 4B) to SCs opsonized with IgM anti-GM2 positive patient sera was significantly increased compared to that seen with IgM anti-GM2-negative sera (Fig. 4C). Moreover, FC_IgM_ and FC_C3_ correlated strongly (Fig. 4D).

**Fig. 5:**
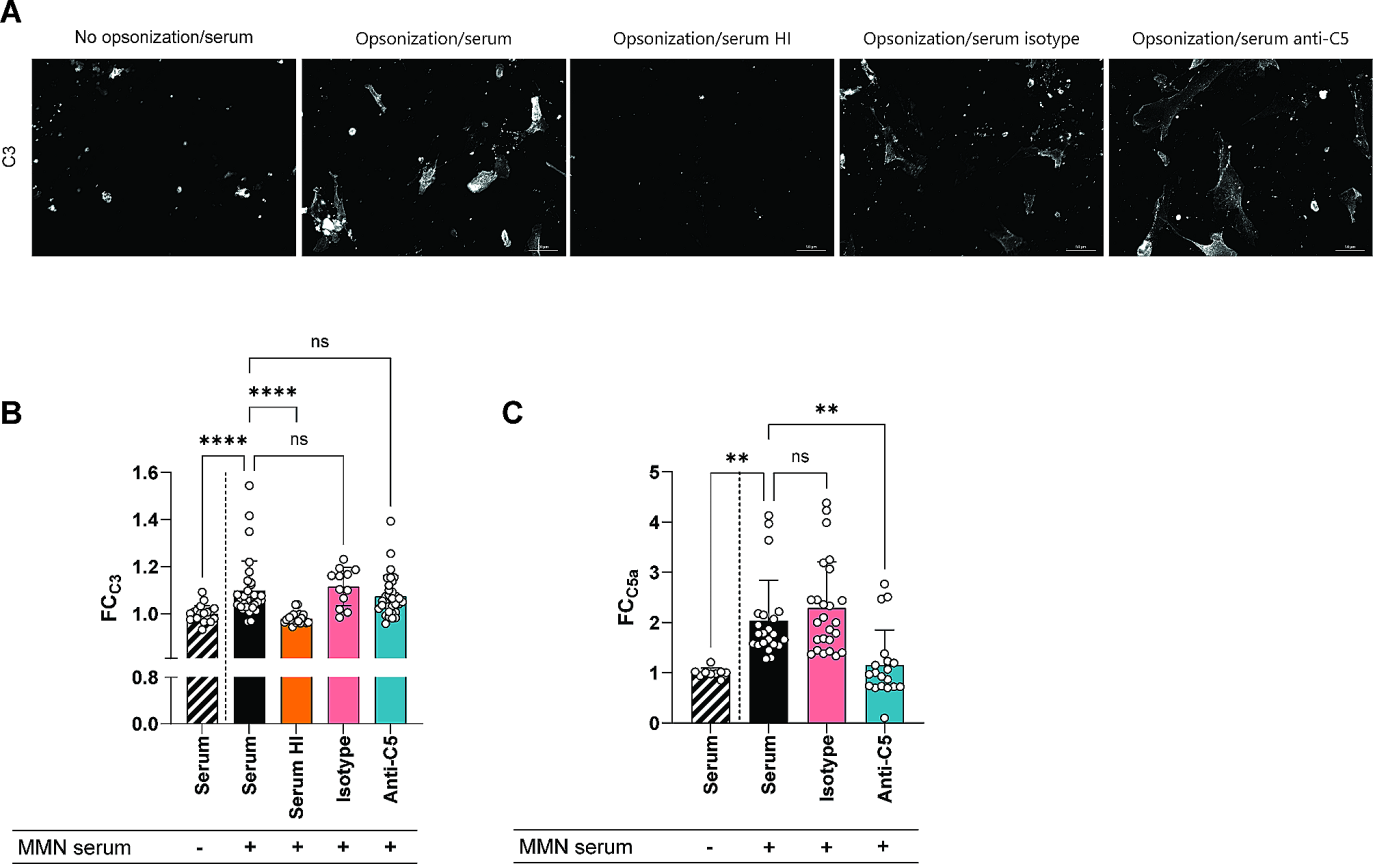
Complement activation by IgM anti-GM2 bound to SCs in culture. (**A**) Representative microscopic images of complement activation on SCs opsonized with IgM anti-GM2 positive MMN serum or not, and incubated with fresh serum, heat-inactivated serum (serum HI), or serum pre-incubated with a monoclonal antibody that blocks C5 activation or an isotypic control antibody, as complement source. 20x magnification, scale bar: 50 μm. (**B**) Quantification of microscopy data using 3 different MMN sera for opsonization.C3 fixation to the cells (quantified as MGV) is expressed as FC (FC_C3_) setting the fixation observed with cells not opsonized with MMN serum (striped bar) as 1. Opsonization of the cells with MMN serum results in a significant increase in C3 fixation, which is abrogated when heat inactivated serum is used as complement source, and which is also reduced wen the complement source is pre-incubated with anti-C5 antibody, and not with an isotypic control antibody. (**C**) Quantification of C5a, depicted as FC_C5a_, measured in the supernatant of SC cultures. C5a generation in culture medium is increased upon opsonization of the cells with IgM anti-GM2 positive MMN patient serum and incubation with fresh serum. This C5a generation is inhibited by an anti-C5 antibody added to the complement source but not by a control antibody. Mean + SD, Kruskal-Wallis, post-hoc Dunn’s multiple comparisons test, ** *p* < 0.01; **** *p* < 0.0001; FC: fold change; HI: heat inactivated; MMN: multifocal motor neuropathy; ns: non-significant

We further assessed complement activation on SCs in culture using membrane-bound and soluble complement activation markers as read-out. We confirmed C3 fixation upon opsonization of SCs with IgM anti-GM2 positive patient sera and complement active serum microscopically. The level of C3 fixation observed on opsonized SCs following incubation with HI serum was similar to the non-opsonized serum control. Both pre-incubation of complement active serum with an irrelevant control antibody or an anti-C5 antibody did not decrease C3 fixation (representative microscopic images depicted in Fig. 5A, quantified as FC to the non-opsonized serum control for multiple experiments in Fig. 5B). To assess down-stream complement activation, we measured C5a in the culture medium of opsonized and complement-exposed SCs. C5a increased upon the addition of complement active serum to opsonized SCs. This increase was significantly inhibited by pre-treating the complement active serum with an anti-C5 antibody which reduced C5a levels to those observed in the non-opsonized serum control (Fig. 5C).

## Discussion

In this study, we show that 10% of 124 patients with MMN have circulating IgM antibodies against the ganglioside GM2, which is mainly expressed on SCs. Disease symptoms of this subgroup of patients, some of whom did not have detectable IgM anti-GM1 antibodies, were indiscernible from those of the other MMN patients, except for an earlier onset of muscle weakness, and the presence of subjective sensory disturbances. Our findings suggest a pathogenic role of IgM anti-GM2 antibodies and involvement of SCs in at least a subgroup of patients.

Gangliosides are a group of sialic acid containing glycosphingolipids that are expressed in the plasma membrane of cells of both the peripheral and central nervous system [11], and by ensuring myelin integrity contribute to optimal saltatory conduction [13]. GM1, GD1a, GD1b and GT1b are the predominant gangliosides in neural tissue. GM1 is localized around the nodal axolemma and nodal Schwann cell membranes. GM2 is less abundant than other gangliosides. On peripheral nerves, GM2 is localized at the abaxonal site of SCs, and to a lesser extent on the abaxonal membranes and the axonal area [20, 27–29]. Here, we show that IgM anti-GM2 antibodies in sera from MMN patients specifically bind to GM2 on a SC line.

GM1 and GM2 are structurally related and differ in only one galactose residue with is added to GM1 during its synthesis from GM2 [30]. Although this structural similarity between GM1 and GM2 raises the possibility of cross-reactivity of IgM anti-GM1 antibodies with GM2, and vice versa [3], our results show that binding is likely to be specific and not cross-reactive. Both in ELISA as well as in the SC model, binding of IgM from most patients with anti-GM2 antibodies was inhibited by pre-incubation with soluble GM2 and not, or only minimally, by soluble GM1. In order to confirm target-specificity, antibody isolation and sequence analysis [31] is key to understand the complex interaction between IgM anti-ganglioside antibodies and their respective targets.

The prevalence of IgM antibodies against GM2 of 10% in our large MMN cohort is in line with the 6–10% found in previous studies [3, 21, 32, 33]. IgM anti-GM2 antibodies have been described throughout the spectrum of immune-mediated neuropathies, including Guillain-Barré syndrome (GBS) and its acute motor axonal variants, chronic inflammatory demyelinating polyneuropathy, and sensory demyelinating neuropathy with ataxia [20, 21, 32, 34–38]. IgG anti-GM2 antibodies are a biomarker for immune-mediated polyneuropathies in cats [39]. Interestingly, the presence of IgM anti-GM2 antibodies is associated with preceding cytomegalovirus (CMV) infection in patients with GBS [40]. The relationship between MMN susceptibility and preceding CMV infections is unknown, but, given the chronic course, not likely.

We found IgM anti-GM2 antibodies to be associated with earlier onset of muscle weakness. Interestingly, IgM anti-GM2 antibodies have been reported in case-reports of children diagnosed with MMN, and in an Indian cohort of childhood-onset GBS [35, 41, 42]. In a selected subgroup of patients with MMN whose samples were included in the complement activation assays (see Fig. 4), the presence of IgM anti-GM2 antibodies was also associated with the presence of subjective sensory complaints but normal sensory nerve conduction studies. Whether sensory complaints in the group with IgM anti-GM2 antibodies are a mere reflection of longer disease duration [4, 23, 24, 43], or reflect a specific pathological effect of IgM anti-GM2 antibodies on sensory neurons remains to be determined. Importantly, patients with MMN with IgM anti-GM2 antibodies had similar disease characteristics, response to IVIg treatment, and disease trajectories as patients without these antibodies, including the 4 patients with only IgM anti-GM2 antibodies, indicating that the presence of IgM anti-GM1 antibodies is not a prerequisite in MMN. This suggests that IgM anti-GM1 and anti-GM2 antibodies trigger a similar pathological mechanism.

Although IgM anti-GM2-induced complement-mediated cytotoxicity has been described previously using a neuroblastoma cell line that expresses GM2 [20, 44], we did not observe lysis of SCs upon complement activation by bound IgM anti-GM antibodies. This is presumably due to the protective effects of membrane complement regulatory proteins, including CD59 [14, 45–47], which we also found to be highly expressed in our SC model [14]. We hypothesize that the contribution of complement activation by IgM anti-GM2 antibodies bound to SCs in MMN pathology is the deposition of other complement components than the membrane attack complex and the production of soluble activation products. We detected C5a generation by IgM bound to SCs in supernatants (Fig. 5). Similar mechanisms may be relevant for MNs, which are also well protected by complement regulatory membrane proteins [14]. Receptors for C3a and C5a are expressed by motor neurons [14] and glial cells [48] and their engagement results in increased inflammation. SCs upon stimulation produce inflammatory cytokines, including IL-1β, IL-6, and TNF-α, which could amplify immune activation and inflammation [49–51]. The importance of crosstalk was suggested in an in vitro model where SCs were activated by neurons upon complement-activation by an anti-GQ1b antibody [52]. Therefore, we postulate that the pathologic mechanism shared by IgM anti-GM1 bound to MNs and anti-GM2 antibodies bound to SCs underlying MMN is the generation and deposition of upstream complement activation products. Generation of these activation products could induce an inflammatory interplay between MNs and SCs resulting in MN dysfunction and thickening of affected nerves.

We acknowledge that the use of two separate cell lines is a limitation of this study, as is the non-myelinating nature of the SC line, since this could affect the ganglioside distribution in comparison to myelinated nerve tissue. Additionally, there could be a difference in overall ganglioside distribution between the sNF96.2 Schwann cell line and Schwann cells in patients. Therefore, it is important to ultimately reproduce some of the key findings using primary SCs or nerve tissue. Nevertheless, the current model allowed us to investigate anti-ganglioside antibody interactions and subsequent immunological effector mechanisms in more detail than before.

In conclusion, we show that IgM anti-GM2 antibodies that target SCs are found in 10% of patients with MMN, sometimes in the absence of detectable anti-GM1 antibodies. Anti-GM2 antibodies are associated with a clinical phenotype of MMN that except for an early onset is indiscernible from the disease associated with anti-GM1 antibodies, suggesting a common pathogenic mechanism shared by either type of antibody. We postulate that this mechanism includes the generation of fluid-phase complement activation products that interact with receptors on SCs and MNs.

### Electronic supplementary material

Below is the link to the electronic supplementary material.

Supplementary Material 1

## Data Availability

Anonymized data and documentation of this study will be shared upon reasonable request from any qualified researcher. Standard data sharing agreements apply.

## Abbreviations

AMAN: Acute motor axonal neuropathy
CSF: Cerebrospinal fluid
CMV: Cytomegalovirus
FB: FACS buffer
FC: Fold change
FCS: Fetal calve serum
GBS: Guillain-Barré syndrome
HC: Healthy control
HI: Heat inactivated
iPSC: Induced pluripotent stem cell
IVIg: Intravenous immunoglobulins
MMN: Multifocal motor neuropathy
MRCss: MRC sum score
NCS: Nerve conduction studies
RT: Room temperature
ScIg: Subcutaneous immunoglobulins
SCs: Schwann cells
SNAP: Sensory nerve action potential
VB: Veronal buffer
