# Supplementary material for: IgM anti-GM2 antibodies in patients with multifocal motor neuropathy target Schwann cells and are associated with early onset
Source: J Neuroinflammation. 2024 Apr 17;21:100. doi: 10.1186/s12974-024-03090-y (PMC11025174; doi:10.1186/s12974-024-03090-y)
Supplement: Supplementary file 1 — Supplementary Material 1 [file 12974_2024_3090_MOESM1_ESM.docx]

1. **Supplemental material**

**
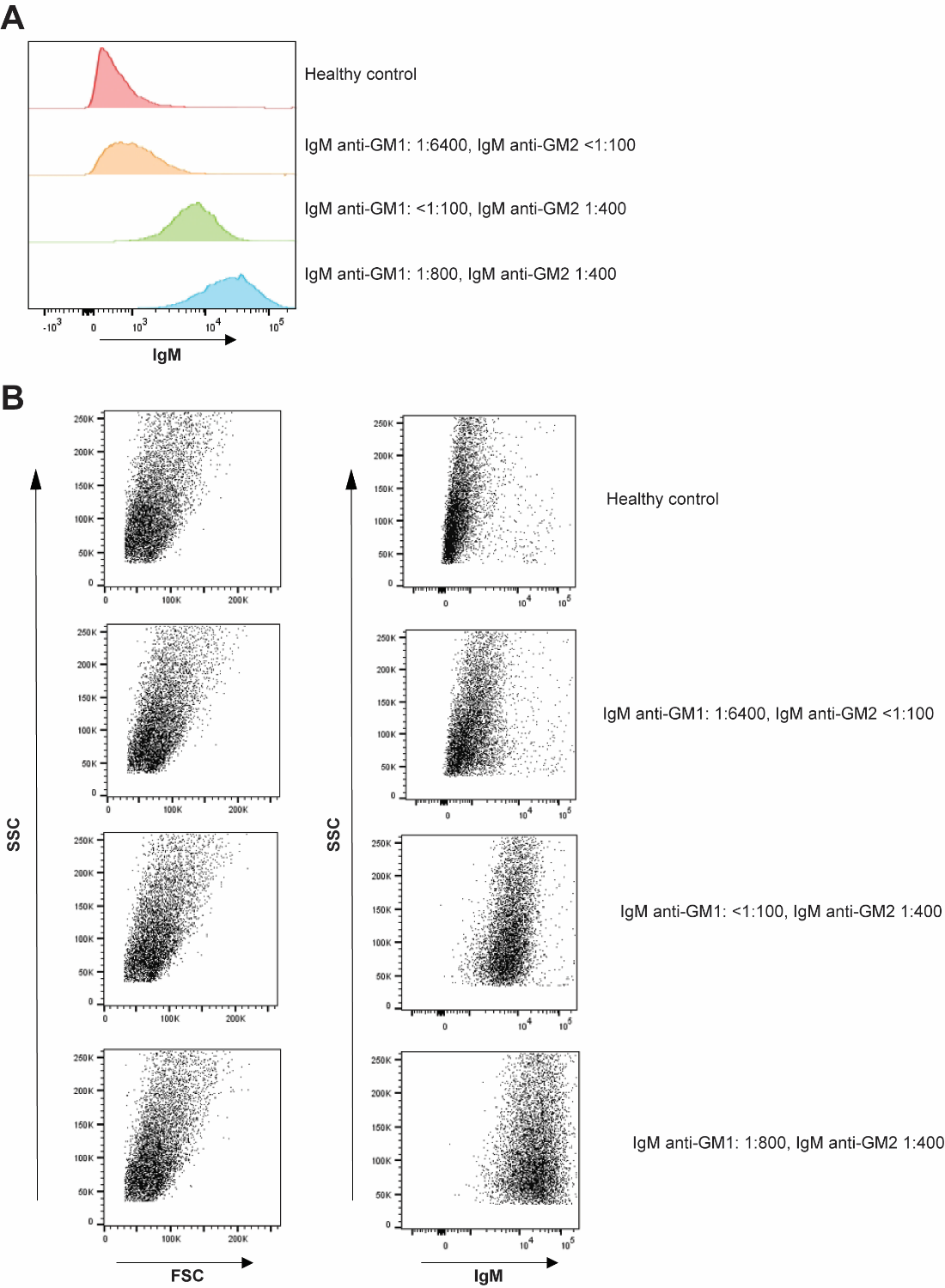
**

**Supplemental Figure 1: Flow cytometry based analysis of IgM antibody binding to Schwann cells**

**A** Schwann cells were incubated with healthy control or MMN patient sera. Respective overview of IgM binding intensities after opsonization with healthy control serum (red), MMN serum positive for IgM anti-GM1 and negative for IgM anti-GM2 (orange), MMN serum negative for IgM anti-GM1 and positive for IgM anti-GM2 (green), and positive for both IgM anti-GM1 and anti-GM2 (blue). **B** Forward scatter (FSC)/sideward scatter (SSC) and IgM/SSC plots of data depicted in **A**.

| **Supplemental Table 1.** Anti-ganglioside antibody titer of MMN patients | | | | |
| --- | --- | --- | --- | --- |
| used in complement activation assays | | |  |  |
|  |  |  |  |  |
|  |  |  |  |  |
| **Patient** |  | **IgM anti-GM1 titer** |  | **IgM anti-GM2 titer** |
|  |  |  |  |  |
|  |  |  |  |  |
| MMN-004 |  | 200 |  | 200 |
| MMN-005 |  | 800 |  | 800 |
| MMN-007 |  | 200 |  | 200 |
| MMN-010 |  | <100 |  | 200 |
| MMN-015 |  | <100 |  | 400 |
| MMN-022 |  | 400 |  | 200 |
| MMN-024 |  | 6400 |  | 200 |
| MMN-026 |  | <100 |  | 400 |
| MMN-048 |  | 100 |  | 1600 |
| MMN-076 |  | 800 |  | 400 |
|  |  |  |  |  |
|  |  |  |  |  |
| MMN-002 |  | 12800 |  | <100 |
| MMN-021 |  | 6400 |  | <100 |
| MMN-028 |  | <100 |  | <100 |
| MMN-042 |  | 1600 |  | <100 |
| MMN-045 |  | 1600 |  | <100 |
| MMN-053 |  | 400 |  | <100 |
| MMN-068 |  | 1600 |  | <100 |
| MMN-070 |  | 200 |  | <100 |
| MMN-094 |  | 400 |  | <100 |
| MMN-106 |  | 100 |  | <100 |
|  |  |  |  |  |
